# Supplementary figures and images for: An auditory brain-computer interface based on selective attention to multiple tone streams
Source: PLoS One. 2024 May 23;19(5):e0303565. doi: 10.1371/journal.pone.0303565 (PMC11115270; doi:10.1371/journal.pone.0303565)

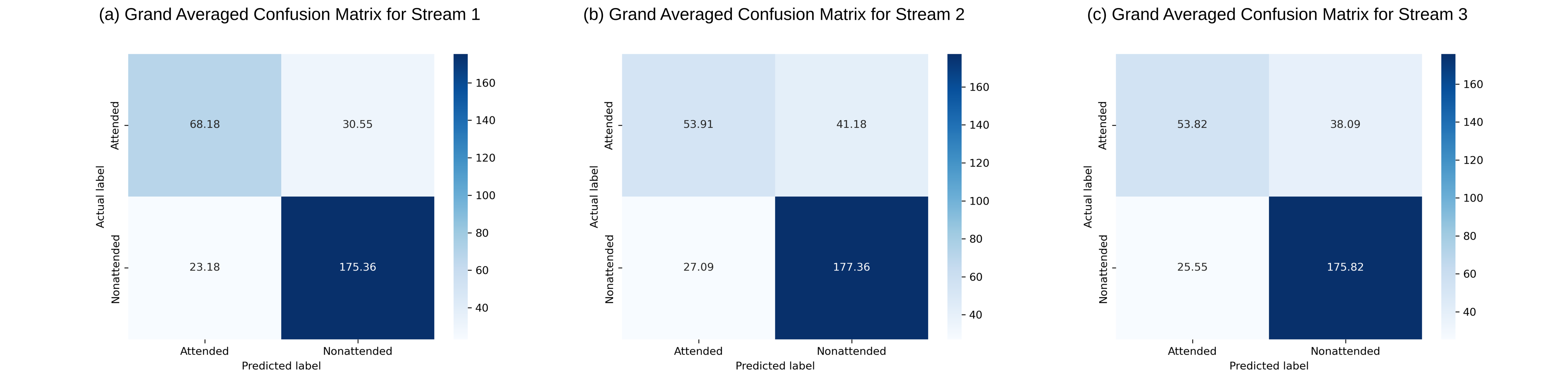

Supplement: S1 Fig — From the classification results from 10-fold cross-validation for each subject, a confusion matrix was derived, and the confusion matrices from all subjects were averaged over subjects. (TIF) [file pone.0303565.s001.tif]
