# Supplementary material for: An auditory brain-computer interface based on selective attention to multiple tone streams
Source: PLoS One. 2024 May 23;19(5):e0303565. doi: 10.1371/journal.pone.0303565 (PMC11115270; doi:10.1371/journal.pone.0303565)
Supplement: Attachment — Submitted filename: Response to reviewers.pdf [file pone.0303565.s002.pdf]

Responses to Editor Comments

1. *Please ensure that your manuscript meets PLOS ONE's style requirements, including those for file naming. The PLOS ONE style templates can be found at https://journals.plos.org/plosone/s/file?id=wjVg/PLOSOne_formatting_sample_main_body.pdf and https://journals.plos.org/plosone/ s/file?id=ba62/PLOSOne_formatting_sample_title_authors_affiliations.pdf*

   Thank you for pointing this out. We used Latex templates, which we can download from the PLOS ONE website. We believe it meets the style requirement; however, if it's not, please let us know.

2. *Please note that PLOS ONE has specific guidelines on code sharing for submissions in which author-generated code underpins the findings in the manuscript. In these cases, all author-generated code must be made available without restrictions upon publication of the work. Please review our guidelines at https://journals.plos.org/plosone/s/materials-and-software-sharing#loc-sharing-code and ensure that your code is shared in a way that follows best practice and facilitates reproducibility and reuse.*

   We will upload the source code used in the analysis and the measurement data. In the first submission, we noted that we will publish our data on Dryad. However, we decided to upload it to HARVARD Dataverse. We have noted the new data sharing statement in the cover letter.

3. Thank you for stating the following in the Acknowledgments Section of your manuscript: "This work was supported by JSPS KAKENHI Grant Number JP23K11811" We note that you have provided funding information that is not currently declared in your Funding Statement. However, funding information should not appear in the Acknowledgments section or other areas of your manuscript. We will only publish funding information present in the Funding Statement section of the online submission form. Please remove any funding-related text from the manuscript and let us know how you would like to update your Funding Statement. Currently, your Funding Statement reads as follows: "..." Please include your amended statements within your cover letter; we will change the online submission form on your behalf.

   We have removed the funding statement from the manuscript and noted a new statement in the cover letter.

4. We note that you have stated that you will provide repository information for your data at acceptance. Should your manuscript be accepted for publication, we will hold it until you provide the relevant accession numbers or DOIs necessary to access your data. If you wish to make changes to your Data Availability statement, please describe these changes in your cover letter and we will update your Data Availability statement to reflect the information you provide.

   We will upload all recorded data.

5. Please include captions for your Supporting Information files at the end of your manuscript, and update any in-text citations to match accordingly. Please see our Supporting Information guidelines for more information: http://journals.plos.org/plosone/s/supporting-information.

   Since we added a figure in supporting information section, the captions for it is included in manuscript.

Responses to Reviewer 1

We appreciate your insightful and constructive feedback on our manuscript. We incorporated all comments and believe the quality of the paper has improved a lot. Here, we have noted the responses to your comments.

1. the number of subjects is small and without any testing on patients. It is therefore strongly recommended to recruit some more subject, if not patients, given the relatively simple paradigm.

   Thank you very much for your comments. This paper we submitted was intended to evaluate the feasibility of the BCI paradigm by presenting three streams consisting of musical tones, and we did not conduct experiments on patients. However, as you pointed out, our paradigm is relatively simple, and we have added three additional subjects in this revision to increase the reliability of the reproducibility of our results.

2. The authors mentioned auditory BCIs for ALS, but missed some key publications in this field, such as 10.3389/fnins.2017.00251, and 10.1007/978-981-16-5540-1_34.

   We cited the publication you suggested in the sentence that mentioned BCI for ALS patients. (line 4)

3. did any of the subjects have experience with auditory BCI? and if there was a testing session where the subjects can learn the paradigm?

   We had no subject who had a long-term experience with auditory BCI. All subjects spent 15-20 minutes before starting the task to familiarize themselves with the paradigm, listen to the stream, and practice the task. We also mentioned in the manuscript that there was time for familiarization to clarify this point. (lines 117 - 119)

4. the image quality needs to be improved, as they look blurred in the pdf

   A PDF file compiled by PLOS ONE shows only a low-resolution preview image, and the link at the top of the page allows you to download a high-resolution image. All original figures are available with higher resolution. Please download them from the links in the PDF.

   "*The compiled submission PDF includes low-resolution preview images of the figures after the reference list. The function of these previews is to allow you to download the entire submission as quickly as possible. Click the link at the top of each preview page to download a high-resolution version of each figure. Links to download Supporting Information files are also available after the reference list.*"

   Ref: https://journals.plos.org/plosone/s/submission-guidelines

5. it is recommended to report the accuracy from each stream, so as to show if there is any imbalance among streams.

   The classification scores noted in Table 2 show accuracy from all streams. Also, we added Fig 6, which shows scores for each subject and streams with a barplot for better visualization.

Responses to Reviewer 2

We appreciate your insightful and constructive feedback on our manuscript. We incorporated all comments and believe the quality of the paper has improved a lot. Here, we have noted the responses to your comments.

Abstract section:

1. Author needs to mention the selected EEG features to achieve the classification accuracy > 80% for 3 subjects and > 75% for 4 subjects. Author needs to explain why the accuracy drop to 75% for 4 subjects.

   In the abstract, we have discussed the reasons for the difference in classification accuracy among subjects.

Introduction section:

1. Author had mentioned in this section , "The largest amplitude is observed on the vertex" (page 2/9). Author needs to explain this statement. What is the value of the amplitude?

   These sentences were for a brief description of the P300, and we did not intend to explain our data. So we do not have any specific number for this. To clarify this part, we have incorporated the description of P300 (lines 10 - 16).

2. The literature review on the Auditory BCI is good. However, there is only 10 citations from the references in this section which is not enough for the standard of the journal. It is suggested to author to cite more latest references such as in year 2020 till 2023.

   We added 13 articles including newer pulications.

Materials and Method section:

1. It is suggested to author to start this section with the process flowchart of the study.

   A time chart of the experiment is added at the top of this section. (Fig 3)

2. Author needs to create and implement the confusion matrix for the classification process.

   The average confusion matrices are implemented in Supporting Information (S1 Fig). Please let us know if it is essential to include it in the body so we can implement it. In this study, a binary classifier was generated to detect selective attention to each stream. Since we had three streams, three different confusion matrices are implemented.

3. Author needs to explain how the selection of the EEG features are done.

   The process of generating feature vectors from each epoch is described in detail (lines 172-186).

4. It is much preferable if the author can provide the sketch of the EEG montage to show the location of the EEG measurement.

   A figure of the EEG montage used in the experiment is shown in Fig 2.

5. It is good that author had provided the evidence of the work ethic approval.

   Thank you for the comment.

6. The procedure of the statistical analysis is not clearly described.

   We have described the two groups tested by t-test (lines 131-139).

7. Author needs to share the example of the xDAWN filter and explain the procedure of using this filter.

   We have implemented the detailed description of the xDAWN filter (lines 152-158).

8. It is only 2 equations are provided which is for calculating Sample Covariance Matrix (SCM). Author needs to provide formula equation for other processes such as 10-fold cross validation and t-test, and EEG signals features ERP, SSNR, Riemannian geometry and MCC.

   We have implemented these as follows.

   - 10-fold cross validation: lines 186-192
     - Since we used three binary classifiers for each of the three streams, we could apply 10-fold cross validation for each classifier. To clarify this point, we have described how it was classified by binary classifier.
   - XDAWN filter including SSNR: lines 152-158
     - We have added brief description of xDAWN filter which maximize SSNR of ERPs.
   - Riemannian Classifier: lines 172-186
     - We have implemented equations used for generating feature vectors.
   - MCC: lines 194-197
     - The description of MCC is added.
   - t-test: lines 131-139
     - We have described the two groups tested by t-test.

9. Author needs to verify this statement, "Recorded signals were bandpass filtered at 1 Hz to 40 Hz (FIR filter) at page 4/9. It is contradicted with the previous paragraph where author have mentioned that "Recorded signals were bandpass filtered at 1 Hz to 40 Hz (2nd Butterworth filter). It is because Butterworth filter IS NOT FIR filter.

   In this study, Matlab was used for visualization and Python for classification. Therefore, the analysis pipelines were different. A Butterworth filter was used for visualization, and the classification was done using an FIR filter. However, for greater clarity, we have implemented the Butterworth filter in the classification, re-ran the analysis, and have listed the results. (Table 2)

10. Author needs to defined MCC and share formula of MCC.

    We have implemented the formula of MCC and how the MCC score can be interpreted (line 194-197).

Results and Discussion section:

1. The results are presented in term of the response to target stimuli as shown in Figure 2 and Figure 3 and also classification results in Table 2. The explanation of the figures and table are good. How about statistical results? How about the 10-fold cross validation results? How about the statistical analysis results, t-test?

   The statistical results of ERP are shown in Fig 4 and Fig 5. To clarify this point, we have mentioned that the gray boxes in the figures show the significant difference detected by the t-test (lines 220-222). The classification scores shown in Table 2 are results of 10-fold cross validation.

2. What is the results of the confusion matrix for the classification process?

   The average confusion matrices were implemented in Supporting Information. (S1 Fig)

3. It is suggested to author to represent the results in term of graph for better view and understanding.

   We have implemented a bar plot that shows the classification scores for each stream and subject (Fig 6).

4. Author needs to explain the results of the >80% and >75% classification accuracy.

   A possible explanation for dropping the classification scores for some subjects have discussed. (lines 253-262)

Acknowledgement section:

1. Good.

   Thank you for the comment. The editor pointed out that we cannot mention our funding information in the Acknowledgement section. Hence, we removed the statement and moved it to the designated section.

References section:

1. 18 references are not good enough since the manuscript will be sent to Journal. It is suggested to author to cite more than 30 papers including the latest paper, such as published in year 2020 till 2023.

   We added 13 articles, including the latest work.

2. There are very old references which are reference # 2 (published in year 1988), reference # 9 (published in year 1990).

   We cited reference #2 to introduce the first P300 speller study by Farwell and Donchin. Therefore, we have also cited some latest papers related to this work. Moreover, reference #9 is a book used as a comprehensive textbook in the study field of auditory scene analysis. We also cited the newer research in this field.

List of changes in this revision

Overall:

1. The number of subjects were increased from eight to eleven.
2. Fig.4, Fig.5 was updated, since the number of subjects was increased.

Abstract:

1. The possible reason for dropping the classification accuracy was discussed.

Introduction:

1. Some publications were cited.
2. The brief explanation of ERP and P300 was added. (lines 10 – 16)

Materials and methods:

1. The figure of EEG montage was added. (lines 104-105)
2. The time chart of the EEG experiment was added. (lines 117-119)
3. In the first manuscript, we mentioned the filter range for visualizing ERP was 1-40Hz, but it was incorrect, so we corrected it to 0.1-40Hz. (line 124)
4. The detailed explanation of t-test was added. (lines 131 – 139)
5. The filter type for classification pipeline was changed from FIR filter to 2nd-order Buttorworth filter. (line 142)
6. The explanation of xDAWN was added. (lines 152 – 158)
7. The explanation of Riemannian classifier was added. (lines 172 -186)
8. The explanation of 10-fold cross validation was added. (lines 186 – 192)
9. The explanation of MCC was added. (lines 194 – 197)

Results and Discussions:

1. It was mentioned that the gray boxes in the ERP plots are results of t-test. (line 220)
2. In Table 2, we listed the precision scores instead of accuracy by mistake. We corrected it. Also, all the classification scores were updated since the type of filter was changed, as mentioned above.
3. Accuracy drop for some subjects was discussed. (lines 253 – 262)
4. The confusion matrices were implemented. (lines 265 – 267)
